# The influence of a light and dark cycle on the egg laying activity of *Aedes aegypti* (Linnaeus, 1762) (Diptera: Culicidae)

**DOI:** 10.1590/0074-02760170362

**Published:** 2018-02-05

**Authors:** Luana Cristina Farnesi, Christine Silveira Barbosa, Luciana Ordunha Araripe, Rafaela Vieira Bruno

**Affiliations:** 1 Fundação Oswaldo Cruz-Fiocruz, Instituto Oswaldo Cruz, Laboratório de Biologia Molecular de Insetos, Rio de Janeiro, RJ, Brasil; 2 Instituto Nacional de Ciência e Tecnologia em Entomologia Molecular, Rio de Janeiro, RJ, Brasil

**Keywords:** oviposition behaviour, mosquito eggs, egg laying preference, light/dark cycles

## Abstract

**BACKGROUND:**

The epidemiological importance of the mosquito *Aedes aegypti* as a vector of multiple human pathogens has generated a growing number of studies on the physiology and behaviour of its blood-feeding females. The activity of oviposition is one of the critical elements contributing to the expansion of *Ae. aegypti's* populations. Although there is a vast literature about oviposition behaviour, significant specific knowledge about egg viability and female fertility under light and dark conditions is still lacking.

**OBJECTIVES:**

We studied, in controlled laboratory conditions, the effect that light and dark cycles have on the efficiency of oviposition by *Ae. aegypti* females.

**METHODS:**

Physiological assays were performed using synchronised eggs obtained from forced egg laying. The number and viability of eggs was analysed under three different light/dark regimes: LD12:12 (12 h of light and 12 h of dark), DD (constant darkness) and LL (constant light).

**FINDINGS and CONCLUSIONS:**

Our results show that females prefer to lay their eggs in dark conditions, but maximising the number and viability of eggs requires the occurrence of a light/dark cycle. Ongoing research on this theme has the potential of contributing to the proposition of new strategies for control based on the failure of egg laying and hatching.

Every organism on Earth is subjected to daily oscillations of light, temperature, and/or humidity. These cycles have an ongoing influence on the biological functions of an organism during its lifetime. In fact, organisms have adapted to environmental cycles throughout their evolutionary history and display rhythmic variation in physiological and behavioural traits in accordance with these cycles, even when the environmental cues are removed (Marques & Menna-Barreto 1999). A pattern of rhythmic variation in these traits, recurring in periods close to 24 h, is called a circadian rhythm (Marques & Menna-Barreto 1999).

The circadian rhythm described by a certain trait can be a property of the species, and yet it can vary according to life stage, gender, and physiological condition (Marques & Menna-Barreto 1999). Environmental factors that indicate the hour of the day have an entraining role on these rhythms, adjusting their oscillation to a regular period of 24 h (Marques & Menna-Barreto 1999, Kreitzman & Foster 2005). Besides the light/dark and warm/cold cycles that happen within one day, food availability and social interactions may also entrain the clock (Marques & Menna-Barreto 1999).

The circadian clock of the fruit fly *Drosophila melanogaster* (Meigen, 1830) is the most well-known and is often used as a model when characterising the circadian clock of other dipteran species, such as vectors of parasites causing tropical diseases, including *Lutzomyia longipalpis* (Lutz and Neiva, 1912), *Aedes aegypti* (Linnaeus, 1762), and *Culex quinquefasciatus* (Say, 1823) (Meireles-Filho et al. 2006, Gentile et al. 2009). The circadian clock of the mosquito *Ae. aegypti* has been studied in more details during the last decade (Gentile et al. 2009). It has been found that most of the genes working on the endogenous regulation of the clock in *Drosophila* are also present in *Ae. aegypti*, although the functions of all of them have not been elucidated in the latter.

In the mosquito *Ae. aegypti*, behavioural aspects, such as locomotor activity, feeding, and mating, were shown to have circadian patterns of variation (Clements 1999, Lima-Camara et al. 2014). However, we are still missing information on variations caused by light influence of important fitness functions, such as oviposition light/dark preferences and egg viability under different light conditions.

Oviposition sites vary among insect species, because the female's site choice may be based on different factors, e.g. substrate composition, presence of immature of the same species, attractive odour, humidity, and light intensity (Clements 1999, Honório & Lourenço-de-Oliveira 2001, Wong et al. 2011). Females of *Ae. aegypti* tend to oviposit in more than one site within the same oviposition window, which may strategically increase the chances of young surviving and dispersing when ideal oviposition sites are not found (de Abreu et al. 2015). Noteworthy, *Ae. aegypti* also counts on another important strategy for survival: their eggs can resist desiccation. This capacity, called quiescence, prevents the developing embryos from losing water when the substrate becomes dry. Quiescence can last for several months until conditions turn favourable again (Christophers 1960, Consoli & Lourenço-de-Oliveira 1994, Rezende et al. 2008). Both strategies are of great epidemiological importance, because they contribute to the expansion of *Ae. aegypti*'s populations and hence, to the spread of diseases transmitted by this vector (Edman et al. 1998). It is known that *Ae. aegypti* females choose the container breeding mosquito according to biotic and abiotic factors (Wong et al. 2011). Thus, the investigation of how external factors influence the behaviour and physiology of oviposition is extremely relevant to both vector tracking and control. Many ways of monitoring the vector population possible by ovitraps that collect eggs (Romero-Vivas & Falconar 2005). On the other hand, the application of larvicidal requires evaluation regarding the capacity of this breeding site for the *Ae. aegypti* mosquito. The choice of a container breeding mosquito by a female is important to test insecticide efficiency and efficacy, in addition to directing the main focus of their application. Thus, knowledge of the female's preference for not only shaded, but also completely dark places is a facilitator for these strategies, among others.

In laboratory conditions, the peak of oviposition for *Ae. aegypti* has been previously described as occurring in the late afternoon (Haddow et al. 1961, Gomes et al. 2006), whereas in another species of *Aedes* (*Ae. africanus*) (Theobald, 1901) the peak occurs in the mid-afternoon, between 16:00 h-17:00 h, with preference for the light phase (Haddow et al. 1961). However, Gomes et al. (2006) described other peaks of *Ae. aegypti* oviposition during scotophase (19:00 h and 21:00 h). Intriguingly, forced oviposition performed in our laboratory routine experiments has shown that a greater number of eggs were laid when females were in dark conditions (unpublished observations).

Herein, we investigated the influence that light and dark cycles have on the efficiency of oviposition by females of the mosquito vector *Ae. aegypti*. We compared the number and viability of eggs when females were allowed to oviposit under different combinations of light and dark regimes.

## MATERIALS AND METHODS

*Mosquito maintenance* - All experiments were conducted with mosquitoes from laboratory colonies of *Ae. aegypti* (Rockefeller strain) (Kuno 2010), continuously maintained for 17 years by the Laboratório de Fisiologia e Controle de Artrópodes Vetores (LAFICAVE), Instituto Oswaldo Cruz, Fiocruz, Rio de Janeiro, Brazil. Eggs were stored attached to dried filter paper and were brought to hatching according to Farnesi et al. (2009). Mosquitoes were synchronised from larvae to adults to 12 h of light and 12 h of dark (LD), under constant temperature (25°C) and humidity between 40-80%, as described in Rezende et al. (2008).

*Synchronised egg laying under different light regimes and analysis of the efficiency of oviposition* - Five days after adult emergence, 300 inseminated females were collected for blood feeding on anesthetised lab guinea pigs (CEUA-FIOCRUZ LW-20/14) for 20 min. Following blood feeding, 120 fully engorged females were selected and distributed one-by-one in Petri dishes (90 or 150 mm diameter) bedded with filter paper (Whatman No. 1). Petri dishes with females were placed in three light assay treatments (40 females per light regime) for three days, such that oogenesis could occur under the experimental light regime: 1) LD 12:12 cycle - 12 h light/12 h dark; 2) DD cycle - 24 h dark; and 3) LL cycle - 24 h light. Note that in the constant dark (DD) and constant light (LL) regimes, the 12 h equivalent to the day hours in the LD training were called the Subjective Day (Day S) and the 12 h equivalent to the night hours in the LD training were called Subjective Night (Night S). After three days of entrainment in each treatment, 4 mL filtered water was added to each Petri dish to induce oviposition (Farnesi et al. 2009). In every regime, 20 selected females (of 40 females that blood fed for each light condition: LD, DD, or LL) received the oviposition stimulus at the beginning of the light phase (Day or Day S), or received the 4 mL at the beginning of the dark phase (Night or Night S). All females were allowed to oviposit for six hours, after which they were discarded. Then, the eggs were kept in an LD regime until the end of embryogenesis. Three experiments were performed for each light regime.

Eggs were visualised and counted under a stereomicroscope, Stereo Discovery V.12 (Zeiss). The number of eggs per female and the number of females that did not lay eggs were calculated. The methodology of synchronised oviposition was adapted from Rezende et al. (2008).

*Analysis of egg viability* - The method for quantifying egg viability was adapted from Farnesi et al. (2009). In brief, eggs from the three replicates of each light regime cited above were randomly picked and accommodated in Petri dishes previously bedded with a moist Whatman No. 1 filter paper. For each light regime, a total of 150 eggs, split into three independent replicates (50 eggs each), were used. A solution of 150 mg/mL yeast extract was added as a hatching stimulus. Petri dishes with eggs were maintained in constant temperature inside an incubator (25 ± 1°C). Relative humidity inside the incubator ranged from 40% to 80%.

*Statistical analyses* - The oviposition parameters (i) number of eggs per female, (ii) number of females not laying eggs, and (iii) egg viability (percentage of hatching eggs), were compared among phases of each light regime and among light regimes. In all cases, we first performed the Shapiro-Wilk normality test. Pairwise comparisons of the number of eggs per female and egg viability among different light regimes were performed with Wilcox-on-Mann-Whitney tests. For egg viability analysis, comparisons among different light regimes were made with Kruskal-Wallis test followed by Dunns test. Pairwise comparisons of the percentage of females not laying eggs required the use of contingency tables and Fisher exact tests. All statistical analyses were performed with the software *GraphPad Prism 5* (Graphpad Software, Inc). Graphical representation of results was performed with the software *GraphPad Prism 5* and *Excel*.

## RESULTS

**Fig. 1:**
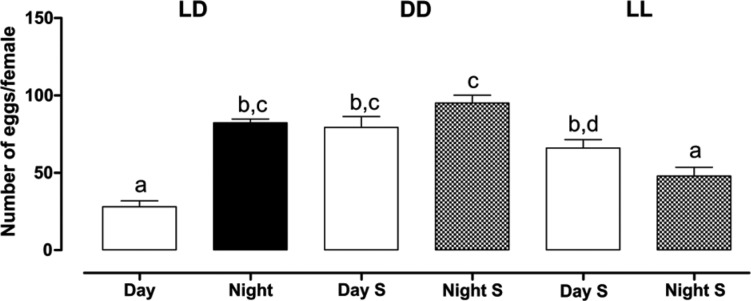
number of eggs per female of *Aedes aegypti*, in different light regimes: LD (Light/Dark), DD (constant darkness), and LL (constant light). Day S (Subjective Day) represents the 12-hour equivalent to the day hours in regime LD and Night S (Subjective Night) represents the 12-hour equivalent to the night hours in regime LD. The bars represent the average of each treatment, and the interval above the bars represents the standard error. Light treatments were compared by Kruskal-Wallis test with Dunns *a posteriori* test (p < 0.0001). Pairwise differences are shown by different letters.

The results of comparing the number of eggs per female between phases, within the light regimes, are shown in Fig. 1. Under the regime LD12:12, the number of eggs per female was significantly higher in the dark phase (82.2 ± 2.56) in comparison with the light phase (28.1 ± 3.85; Mann-Whitney = 302.5, p < 0.05). This difference was not apparent when comparing the subjective day (79.4 ± 6.96) and the subjective night (95.1 ± 5.12) of the constant darkness regime (DD) (Mann-Whitney = 302.5, p = 0.13). On the other hand, egg laying in a regime of constant light (LL) showed that females laid significantly more eggs during the subjective day (66.0 ± 5.44) than during the subjective night (47.9 ± 5.67 eggs, Mann-Whitney = 1369, p < 0.05).

Comparisons among treatments showed that, in general, the number of eggs per female was significantly higher when egg laying happened in the dark (Fig. 1). Significantly fewer eggs were laid when females were stimulated either at the light phase of LD12:12 or during the subjective night of regime LL (Fig. 1). Intriguingly, egg laying in the subjective day of regime LL was not significantly lower than that seen for all dark phases of all regimes (Fig. 1), suggesting that another factor might be in play in this case, because the presence of light did not inhibit egg laying.

**Fig. 2:**
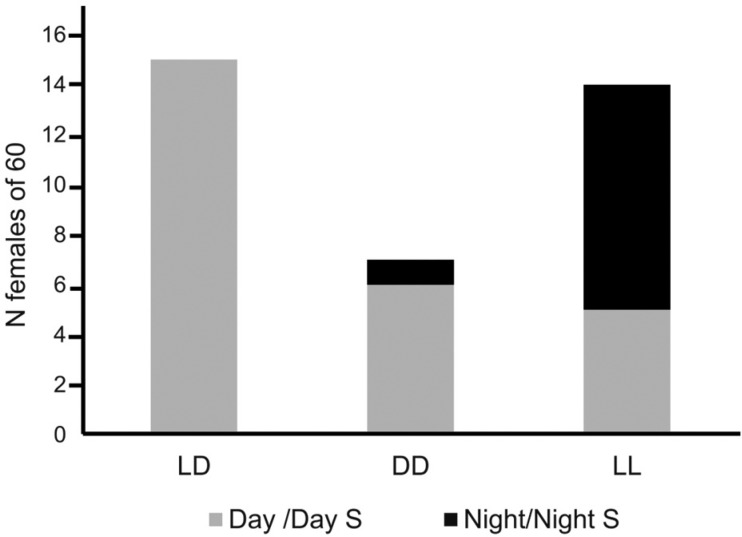
number of females not laying eggs, out of 60 females kept in different light regimes: LD (Light/Dark), DD (constant darkness), and LL (constant light). Day S (Subjective Day) represents the 12-hour equivalent to the day hours in regime LD and Night S (Subjective Night) represents the 12-hour equivalent to the night hours in regime LD. Results from different light regimes were submitted to pairwise comparisons using the Fisher exact test (see results section).

The number of females not laying eggs (out of 60 females), in each light regime, is shown in Fig. 2. The results showed that the light phase of a LD12:12 regime had the largest number of females not laying eggs (15 females, or 25%), whereas all females laid in the dark phase of LD12:12. Although fewer females did not lay eggs, the subjective days of regimes DD (six females, or 10%) and LL (five females, or 8.3%) did not differ significantly (Fisher exact test: p = 0.064), however, considering the p value, a trend could be occurring. Finally, the number of females not laying eggs in regimes LD 12:12 and LL were significantly different (Fisher exact test: p < 0.001). Overall, these results indicate that females prefer laying in dark conditions, but when a light/dark cycle is absent most females lay their eggs regardless of the immediate light condition.

**Fig. 3:**
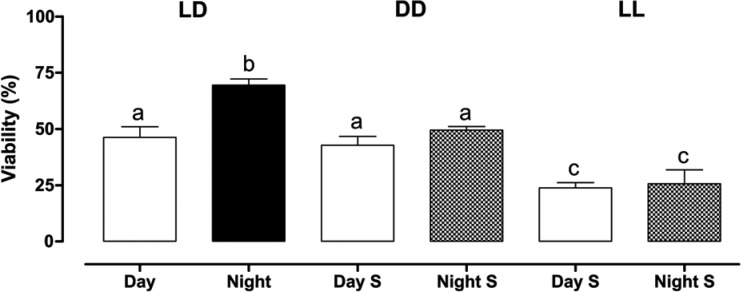
egg viability, calculated as the percentage of hatching eggs for females kept in different light assays. LD (Light/Dark), DD (constant darkness), and LL (constant light). Day S (Subjective Day) represents the 12-hour equivalent to the day hours in regime LD and Night S (Subjective Night) represents the 12-hour equivalent to the night hours in regime LD. The bars represent the average of three replicates, and the interval above the top represents the standard error. Light treatments were compared by Kruskal-Wallis test with Dunns *a posteriori* test (KW = 88.86; p < 0.0001). Pairwise differences are shown by different letters.

The percentage of hatching eggs from each light regime is shown in Fig. 3. Oviposition during the dark phase of regime LD12:12 granted the highest percentage of hatching eggs (69.5 ± 2.72). Meanwhile, eggs laid in both the subjective day and subjective night of LL regime showed the lowest hatching percentage (23.8 ± 2.45 and 25.6 ± 6.25, respectively). Even though more eggs were laid when females were in the dark, as shown in Fig. 1, only approximately 50% of eggs laid in both subjective phases of the DD regime hatched, which did not differ significantly from the percentage hatched when females laid in the light phase of regime LD12:12 (42.3 ± 3.95 in Day S and 49.5 ± 1.59 in Night S). This result strongly suggests that the present of the light condition is not as important as the occurrence of a light/dark cycle for guaranteeing egg viability.

## DISCUSSION

The mosquito *Ae. aegypti* is one of several species of the family Culicidae that are vectors of human pathogens. Among the most important human diseases caused by viruses are Dengue and Urban Yellow Fever, both transmitted by *Ae. aegypti*. Also transmitted by this species of mosquito, the viruses Chikungunya and Zika have been severely affecting human populations over the last 3-5 years, mainly in South America (Valle et al. 2015). Antiviral therapies for these diseases are currently unavailable or inaccessible by most of the population. Thus, strategies for vector control are the best alternatives to restrict the spread of these viruses (Valle et al. 2015). *Ae. aegypti* vector control is primarily directed against larval or adult stages. Historically, mosquito eggs have not been the focus of attention for investment in control, despite the potential of being a relevant control target signalised since the eighties. *Ae. aegypti* eggs can remain viable under dry conditions for months because of egg desiccation resistance, a phenomenon largely studied over the last few years (Rezende et al. 2008). Because of this phenomenon, the eggs have the important capacity of passive dispersal, a mechanism that allows spreading the eggs of this species around the world (Reiter & Sprenger 1987).

In mosquito vectors, the egg is the only stage that does not have the capacity of active dispersion (Reiter & Sprenger 1987). This implies that females need to choose an appropriate oviposition site to ensure the right conditions for survivorship and development of their eggs. The female's choice is based on the perception of specific physical and chemical stimuli from the site (Navarro-Silva et al. 2009). In this sense, the attraction of gravid females for these kind of stimuli, such as a hay infusion or the presence of certain plants, are used in many studies that require egg collection (Consoli & Lourenço-de-Oliveira 1994, Allan & Kline 1995, Wong et al. 2011). Environmental stimuli such as the intensity of light may also influence the site's choice. In the *Aedes* genus, for instance, it is known that eggs and immature stages are more easily found and have better productively in shaded sites (Honório & Lourenço-de-Oliveira 2001, Maciel-de-Freitas & Lourenço-de-Oliveira 2011). Because *Ae. aegypti* females usually spread their eggs in varied oviposition sites (Christophers 1960, Clements 1992), here we assume that confining females for oviposition would be the best method for maximising the number of eggs in a short time (6 h) and on the same site. Indeed, following this method, we acquired enough eggs to allow comparison of oviposition efficiency among the different light regimes.

Blood-feeding mosquitoes, like *Ae. aegypti* females, depend on blood ingestion for the development and maturation of eggs. In fact, egg production is proportional to the amount of blood ingested (Christophers 1960), making it essential to carefully select females with a high volume of blood in their abdomens for the experiments. It is also known that larger females will lay more eggs (Farjana & Tuno 2013). Here we found that, in controlled laboratory conditions, females laid, on average, more eggs in the dark when forced to use a 6 h window of time for oviposition. This result partially corroborates data in the literature: on one hand, the investigation of field females of *Ae. africanus*, without a time restriction for oviposition (Gillett & Haddow 1957), showed that the oviposition peak occurred in the mid-afternoon (around 16:00 h-17:00 h); on the other hand, experiments with *Ae. aegypti* females in the laboratory, by the same group, found that the oviposition peak happened at the beginning of the dark phase (Gillett et al. 1961, Haddow et al. 1961, Gomes et al. 2006). This indicates that, in spite of the diurnal locomotor activity of *Ae. aegypti* (Gentile et al. 2009, Lima-Camara et al. 2014), oviposition activity seems to be preferentially nocturnal. These results suggest that oviposition activity is determined mostly by external conditions, once the oviposition is not abolished in LL, as the locomotor activity is (Rivas et al., unpublished observations), although the endogenous element can be considered a weak factor (Gillett et al. 1959).

According to Gillett et al. (1959), the peak of oviposition in *Ae. aegypti* increases when females return to the dark after a light phase, regardless of the period of time they were exposed to light. Our data from the LD regime confirms that observation.

Our results show that females maintained in the constant light regime (LL) may be under severe stress, because they seem to lay eggs immediately after receiving the water stimulus, and as such guarantee the efficiency of oviposition. However, despite the equivalence in the number of eggs laid in LL when compared to the number of eggs laid in DD, the percentage of females not laying was the highest (Fig. 2) and hatching was the lowest in LL (Fig. 3), showing that females cannot guarantee survivorship of eggs in constant light. This is the first-time egg viability has been investigated in experiments with females of *Ae. aegypti* under different light regimes. Our data indicate that the stress caused by the lack of shading (LL) is more harmful than the stress caused by the lack of light (DD). This is in agreement with the literature on the behaviour of gravid females of species, which seek shaded places for oviposition (Honório & Lourenço-de-Oliveira 2001, Maciel-de-Freitas & Lourenço-de-Olivei-ra 2011). The negative effect of a constant light regime may result from the immediate stress caused by females seeking a shaded site for oviposition, but most likely, from the harm the three-day exposition to constant light had on egg development, before the oviposition stimulus was added. Nevertheless, as shown in Fig. 3, the exposition to constant darkness was also unfavourable for the viability of eggs, as only approximately 50% of eggs hatched when laid by females submitted to this regime.

Overall, the results on egg viability reveal that a light/dark cycle is critical for assuring the hatching of most eggs. Understanding the females’ preference for laying eggs in a light or dark environment and its effects on egg viability is important for the specific knowledge about the biology of this important arbovirus vector. The egg laying behaviour is related to offspring survival and development, and can contribute to regulating *Ae. aegypti* populations. Our data are relevant and can help in adequate mosquito control strategies, directing control actions based on choice of places for traps designated for egg collection and insecticide application (Romero-Vivas & Falconar 2005).

The occurrence of a light/dark cycle is the natural condition for the majority of biological functions, especially those regulated by the so-called clock genes. The functioning of the endogenous circadian clock (central and peripheral) is synchronised by environmental factors, among which the most important is light (Hardin 2011). As an example, the proper operation of the endogenous central clock of *Drosophila* can be summarised as follows: The molecular regulation of the circadian clock involves three negative loops, where the transcription of several clock genes is controlled by their own protein products. Transcription of the central clock genes *period* and *timeless* is promoted by the transcriptional factor CLK-CYC, which is a heterodimer formed by the protein products of the genes *clock* and *cycle* (Hardin 2011). At night, when the levels of proteins PER and TIM are high, they form a protein complex that migrates to the nucleus and binds to CLK-CYC, impeding this transcriptional factor of reaching the promoter and inducing the transcription of *period* and *timeless*. In the presence of light, the flavoprotein CRYPTOCHROME (CRY) suffers a structural change that provokes the degradation of TIMELESS (TIM) via proteasome (Busza et al. 2004). The low levels of TIM during the day impede the formation of the complex with PER, which allows CLKCYC to again activate the transcription of *per* and *tim*.

As described for *Drosophila*, the role of the flavoprotein CRY (also found in *Ae. aegypti*) on the regulation of the transcription of genes *period* and *timeless*, is light-dependent. Thus, although our research does not give a clear-cut answer for the role of light as an environmental synchroniser of the circadian clock (*Zeitgeber*) acting on the rhythm of oviposition, it is possible that the lack of a light/dark cycle may have disrupted a central regulatory loop of the endogenous clock and affected embryogenesis and oviposition. A more detailed investigation, including the analysis of gene expression of clock genes, may help to elucidate whether there is an endogenous control regulating the oviposition activity.
